# High genetic diversity of the himalayan marmot relative to plague outbreaks in the Qinghai-Tibet Plateau, China

**DOI:** 10.1186/s12864-024-10171-y

**Published:** 2024-03-08

**Authors:** Ying Ma, Pengbo Liu, Ziyan Li, Yujuan Yue, Yanmei Zhao, Jian He, Jiaxin Zhao, Xiuping Song, Jun Wang, Qiyong Liu, Liang Lu

**Affiliations:** 1 Qinghai Institute for Endemic Disease Prevention and Control, Xining, 811602 China; 2 National Key Laboratory of Intelligent Tracking and Forecasting for Infectious Diseases, Chinese Center for Disease Control and Prevention, National Institute for Communicable Disease Control and Prevention, Beijing, 102206 China; 3 College of Life Sciences, WuHan University, Wuhan, 430072 China; 4 Center for Disease Control and Prevention of Chaoyang District, Beijing, 100021 China

**Keywords:** Zoonotic spillover, Marmota himalayana, Population genetics, Yersinia pestis, Plague

## Abstract

Plague, as an ancient zoonotic disease caused by *Yersinia pestis*, has brought great disasters. The natural plague focus of *Marmota himalayana* in the Qinghai-Tibet Plateau is the largest, which has been constantly active and the leading source of human plague in China for decades. Understanding the population genetics of *M. himalayana* and relating that information to the biogeographic distribution of *Yersinia pestis* and plague outbreaks are greatly beneficial for the knowledge of plague spillover and arecrucial for pandemic prevention. In the present research, we assessed the population genetics of *M. himalayana*. We carried out a comparative study of plague outbreaks and the population genetics of *M. himalayana* on the Qinghai-Tibet Plateau. We found that *M. himalayana* populations are divided into two main clusters located in the south and north of the Qinghai-Tibet Plateau. Fourteen DFR genomovars of *Y. pestis* were found and exhibited a significant region-specific distribution. Additionally, the increased genetic diversity of plague hosts is positively associated with human plague outbreaks. This insight gained can improve our understanding of biodiversity for pathogen spillover and provide municipally directed targets for One Health surveillance development, which will be an informative next step toward increased monitoring of *M. himalayana* dynamics.

**Supplementary Information:**

The online version contains supplementary material available at 10.1186/s12864-024-10171-y.

## Introduction

Zoonotic spillover, which is the transmission of a pathogen from a vertebrate animal to humans, presents a global public health burden [1]. Understanding the ecological processes before pathogen spillover is crucial for pandemic prevention and mitigating the burden of infectious diseases but it is incomplete. Novel efforts are being undertaken to clarify how biodiversity conservation can help reduce the risk of zoonotic spillover of pathogens from wild animals, sparking epidemics and pandemics in humans [2]. However, the relationship between biodiversity and diseases is still unclear: whether biodiversity influences infectious disease transmission via an amplification effect or a dilution effect [3]. Genetic diversity, as a dimension that comprises biodiversity, has not yet been studied in the context of biodiversity change and zoonotic disease risk.

Plague, as an ancient zoonotic disease caused by *Yersinia pestis*, has brought great disasters throughout human history [4, 5]. Historically, there have been at least three major pandemics [6], and the third was thought to have originated in southwest China in 1772 and then spread around the world [7, 8]. In China, 12 types of plague foci differentiated based on geographic landscapes, hosts, principal vectors, and *Y. pestis* ecotype characteristics are widely distributed. Among them, the natural plague focus of *Marmota himalayana* in the Qinghai-Tibet Plateau is the largest, covering areas of five provinces, including Qinghai, Tibet, Yunnan, Xinjiang, and Sichuan. It has been constantly active and the leading source of human plague in China for decades [9]. In addition, the Qinghai-Tibet Plateau encompassed the most diverse isolates and was supposed to be the source of *Y. pestis*. Due to the widespread existence of annual outbreaks in animals, human cases occur almost every year in the enzootic region, and the mortality rate exceeds 50%.

*M. himalayana* is a marmot species that lives in short grass steppes and alpine at altitudes between 2700 and 5450 m throughout the Qinghai-Tibet Plateau [10]. As the predominant host of the modern plague focus, it has a pivotal role in the maintenance, transmission, and prevalence of plague and is directly or indirectly responsible for most human epidemics [11]. The first route causing human plague cases in this plague focus was skinning and eating *M. himalayana* [12]. *M. himalayana* is social and lives in multiburrow colonies, which facilitates the transmission of *Y. pestis*[13]. With the development of the economy and transportation, human activity is increasingly and persistently disturbing their habitats [14], which may result in contact and communication between different populations of *M. himalayana*, increasing the risk of plague outbreaks. Thus, understanding the population genetics of *M. himalayana* and relating that information to the biogeography of *Y. pestis* and plague outbreaks is greatly beneficial for the knowledge of plague spillover.

Here, we investigated the role of biodiversity in plague spillover by carrying out a comparative study of plague outbreaks and the population genetics of *M. himalayana* populations on the Qinghai-Tibet Plateau. The objectives of our study were to (i) assess the population genetics of *M. himalayana* and clarify its phylogeographical distributions; (ii) compare the concordance between the biogeography of *M. himalayana* and *Y. pestis*; and (iii) evaluate the relevance of plague outbreaks and marmot population genetics to improve surveillance of this zoonotic disease.

## Materials and methods

### Sample collection

**Fig. 1:**
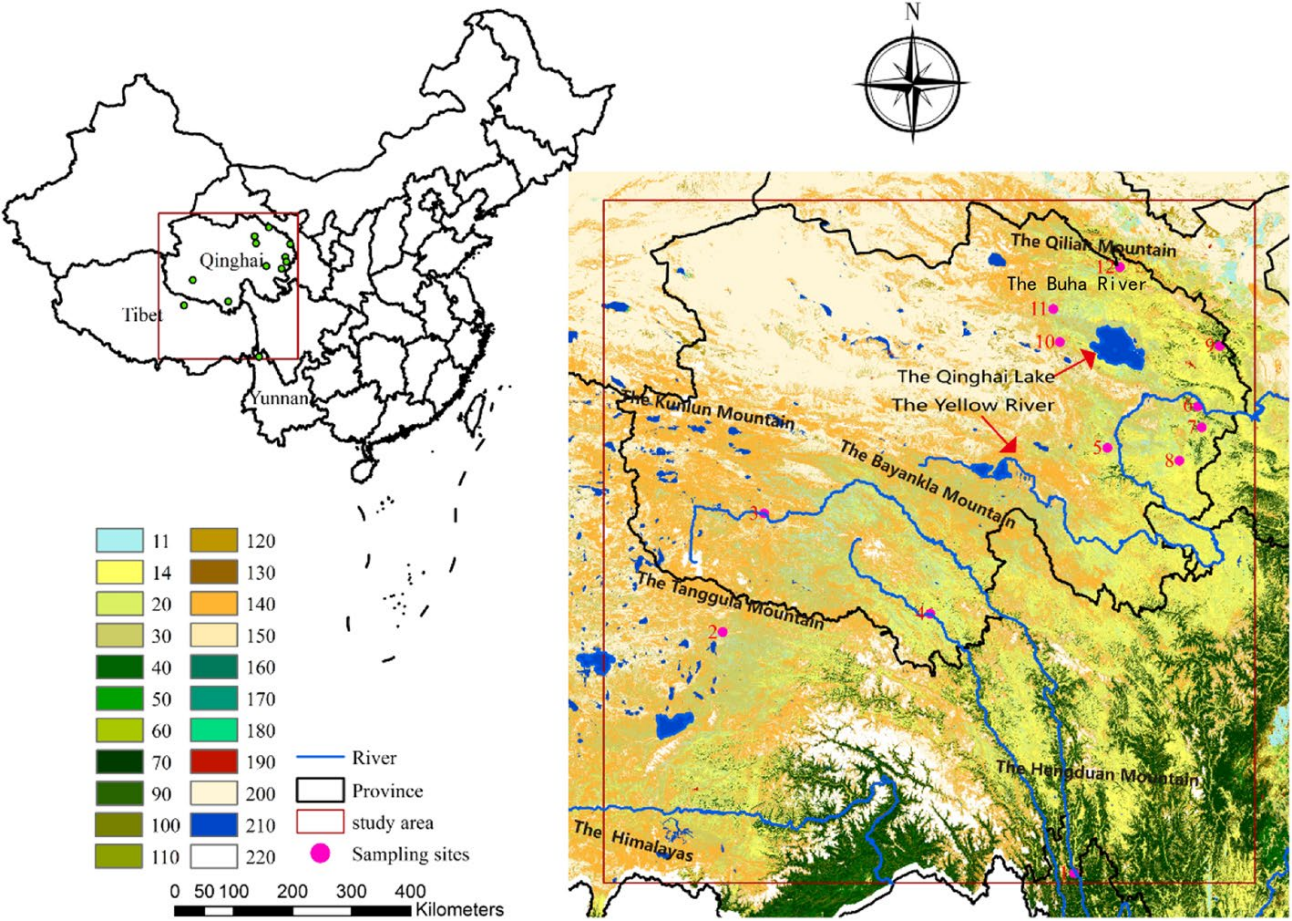
Sampling locations (red blots) in Yunnan (*n* = 1), Tibet (*n* = 1), Qinghai (South- 2, North- 8). ArcGIS 10.3 software (http://www.esri.com/software/arcgis) was used to develop the map. The background represent classification system of land-cover products (GlobCover2009): Post-flooding (11), Rainfed croplands (14), Mosaic cropland 50–70% (20), Mosaic cropland 20–50% (30), Semi-deciduous forest (40), Closed broadleaved deciduous forest (50), Open broadleaved deciduous forest (60), Closed needleleaved evergreen forest (70). Open needleleaved deciduous forest (90), Closed to open mixed broadleaved and needleleaved forest (100), Mosaic forest (110), Mosaic grassland (120), Closed to open shrubland (130), Closed to open herbaceous vegetation (140), Sparse vegetation (150), Fresh or brackish water (160), Saline or brackish water (170), Fresh, brackish or saline water (190), Artificial surfaces and associated area (190), Bate areas (200), Water bodies (210), Permanent snow and ice (220)

A total of 503 M*. himalayana* individuals were collected from 12 counties in Qinghai, Tibet, and Yunnan. These populations represent the overall distribution range of *M. himalayana* in China. All individuals were identified based on morphology. Tissue samples (livers, muscles, or toe clips) were preserved in 95% ethanol for DNA extraction. Relevant sample information regarding sampling sites and sample sizes is shown in Fig. 1 and Table S1. The three-letter abbreviations were used to correspond to the 12 populations of himalayan marmot.

### Laboratory protocols

Total genomic DNA from tissue samples was extracted using a DNAeasy Blood & Tissue Kit following the manufacturer’s protocols. The extracted DNA was stored at -20 °C until further analysis. In this study, 13 microsatellite primers (Table 1) were previously developed for *M. himalayana* []. These primers were labeled with FAM, HEX, and ROX fluorescent, respectively. The final volume of each polymerase chain reaction (PCR) was 25 μl (LA Taq 0.15 μl, dNTP Mix 0.5 μl, 10 × Buffer 2.5 μl, each primer at 0.5 μM, 1 μl of DNA, and 19.85 μl of dd H_2_O). After amplification, the amplicons were checked using a 1% agarose gel electrophoresis method. The PCR product with different dyes was pooled and analyzed with an ABI 3730XL Genetic Analyzer (Applied Biosystems, Foster City, USA), and allele sizes were analyzed using GeneMarker [15].

**Table 1:** Genetic diversity in 12 populations of *M. himalayana* in the Qinghai-Tibet Plateau

Loci	Primer	Marker	Primer Sequence (5’ → 3’)	Annealing Temperature (℃)	Allele size (bp)
B	AHTCA3F	FAM	TTTTTGGCTAACATAGTGGT	55	160
	AHTCA3R		AGTGAAGGCTAAAGCAGAGT		
C	AHTCA30F	HEX	GTCCAAAAAAAAAAGTAAGT	55	194
	AHTCA30R		GAAGTAATTGAACCCATAAA		
D	AHTCDA1F	FAM	ATGGGGACAAACATGGGACT	55	213
	AHTCA1R		CGGTTGCTATGGAGACTGGA		
E	AHTCT37F	FAM	CTTGTTCAGGATTTGGCTAT	56	232
	AHTCT37R		AATGTCTTGAAAATGGTGTT		
G	AHTCT6F	HEX	ATGGCAGAGAATATAAAATGG	55	171
	AHTCT6R		CTGGTGGAACTTGTTAGGAG		
H	AHTCA53F	HEX	GGAAGACCACAGAGGAACAG	56	235
	AHTCA53R		CCTTGAAGAGCAAGAGCATA		
I	AHTCT19F	ROX	TAATATCCCCCAAAGAAGTA	56	194
	AHTCT19R		TAGACCTTGCTGTGAAAAAT		
J	AHTCA90F	FAM	ATGGGACAGAACTCTTGATT	56	226
	AHTCA90R		CCTTATAGTTTTACCTCCTCC		
M	AHTCA49F	FAM	CATTGGAAGACAGAAAATACA	56	181
	AHTCA49R		CAGTCCTTTGAAACTTGAGTA		
R	BHTCT4F	HEX	ACAAAACTTCTTCGTCTC	55	196
	BHTCT4R		GTCTTCCACTACTCCTCT		
S	BHTCA50F	ROX	GTTGATATTCACATACGTTGTA	56	156
	BHTCA50R		CTCACTTGGGATTTGCTT		
T	CHTCA27F-1	ROX	AATAGCCAGTTCAACCTC	55	174
	CHTCA27R		ATGCTAACTTCAGCAACA		
W	CHTCA60F	ROX	TTTCCACAGCAGCACTCT	56	283
	CHTCA60R		GGTTCCTTACCCAGACCA		

Furthermore, two mitochondrial markers (COI and Cytb) were targeted for PCR and sequencing. PCR primers for COI and Cytb of *M. himalayana* were designed with Primer Premier 5.0 software [16] according to the complete gene sequence provided by NCBI (JX069958.1) [17]. Primer sequences for COI and Cytb are shown in Table S2. PCRs were performed using the same ratios of reagents as those used for microsatellite sequencing.

### Microsatellite analyses

Population data sets were screened for null alleles using MICRO-CHECKER [18]. Genetic diversity was characterized with GenAlEx 6.501 [19] by determining the number of different alleles (*Na*), the number of effective alleles (*Ne*), Shannon’s information index (I), observed heterozygosity (*Ho*), and expected heterozygosity (*He*). PIC-Calc 0.6 was used to assess polymorphic information content (PIC). Allelic richness (*r*) and positive inbreeding coefficient (*Fis*) in each population were obtained using FSTAT v 2.9.3 [20]. Linkage disequilibrium (LD) and the possibility of deviations from Hardy–Weinberg equilibrium (HWE) were assessed with Arlequin v 3.5.2.2[21].

To identify the genetic diversification of *M. himalayana*, we performed a series of analyses. First, a Bayesian model-based algorithm implemented in STRUCTURE v2.3.4 was used to infer the number of likely clusters [22, 23]. Six independent runs for each K value (K = 2 to 12) were conducted with a ‘burn-in’ period of 50,000 iterations and 1,000,000 replications. We used Evanno’s method to determine the most likely number of clusters, as implemented in Structure Harvester [23]. The results of 10 replicate runs for each K value were combined using the greedy algorithm of Clumpp 1.1.1[24]. Summary outputs were displayed graphically using District v1.1 [25]. Principal component analysis (PCA) was performed by GenAlex 6.501 [19] in predefined populations to maximize intergroup variation. *Nei’s* standard genetic distance D was used to calculate the unweighted pair-group method with arithmetic averaging (UPGMA) tree through Mega software [26]. Meanwhile, the NJ phylogenetic tree was constructed to measure genetic relationships among populations. The number of putative migrants (*Nm*) per generation between populations was estimated from pairwise *F*_*ST*_, *Nm* ≈ (1-*F*_*ST*_)/4*F*_*ST*_. We examined the partitioning of genetic variation among localities by performing an analysis of molecular variation (AMOVA) using Arlequin.

Genetic variation may be affected by multiple processes, including isolation-by-distance (IBD) and isolation-by-environment (IBE) [27]. We explored the correlation between genetic distance and differentiating factors using two methods: the Mantel test and multiple matrix regression with randomization test (MMRR). The *Fst* value was selected for the genetic distance. In these tests, two different geographical distances were used: Euclidean distance was calculated using the point distance function in the RASTER R package [28], coordinates of sample sites were used to calculate a distance matrix, and the least-cost path (LCP) was implemented using network analysis in ArcGIS [29]. Environmental variables included the 19 bioclimatic parameters, potential evapotranspiration (PET), and elevation. Nineteen bioclimatic parameters and elevations were obtained from the WorldClim database (Table S3) [30]; PET was obtained from the CGIAR Consortium for Spatial Information. First, we extracted environmental variables through ArcGIS (Table S4). Then, a pairwise Spearman correlation analysis was used to exclude pairs of variables with a correlation greater than 0.75 (Table S5). Immediately, a PCA was performed, and the eigenvectors of the first two PCA components were used as environmental variables (Table S6).

### mtDNA analyses

We performed subsequent phylogenetic analysis with the concatenated sequences of COI and Cytb. Haplotype diversity (*h*) and nucleotide diversity (π) were evaluated by DNASP 5.0 [31]. PopART software was used to construct haplotype network graphics through the median-joining method [32]. We inferred phylogenies of the haplotypes with Mega’s maximum-likelihood (ML). We used jModelTest [33] to identify the optimal substitution model as a GTR model by the Akaike Information Criterion (AIC). *Marmot sibirica* was selected as the outgroup species. The fossil calibration point was set as 1.91 Ma according to TIMETREE (http://timetree.org/). For the ML analysis, the significance of each model was tested with 1000 bootstrap replicates. For the Bayes analysis, two independent Markov Chain Monte Carlo (MCMC) analyses composed of four Metropolis-coupled chains each were run for 5,000,000 generations, with trees sampled every 1000 generations. The first 25% of the Markov chain samples were discarded as burn-in, and the chains were checked for stationarity with TRACER v1.7.2 []. The final tree was viewed in FigTree v1.4.0 [34].

To estimate the divergence time of the main mtDNA lineages of *M. himalayana*, we performed a Bayesian MCMC analysis in BEAST 2 [35]. BEAUTI was used to generate the XML formatted input file for BEAST. The strict molecular clock was selected, and a normal distribution was employed for the parameter. The MCMC chains were analyzed for 1,000,000 generations, with sampling every 1000 generations. TRACER v1.7.2 was used to verify the posterior distribution and the effective sample sizes (ESSs) from the MCMC output. TREEANNOTATOR in the BEAST package was used to summarize tree data according to the ‘mean height’, and the first 50% of trees were discarded to represent the ‘burn-in’ period, which ended well after the stationarity of the chain likelihood values had been established. The tree and divergence time were displayed in FigTree v1.4.0. Additionally, we computed the neutrality test (including Tajima’s D and Fu’s Fs) based on the phylogenetic results.

### *Y. pestis* molecular subtyping

Different region (DFR) analysis was used for molecular typing in the plague focus. Information on *Y. pestis* isolates collected on the Qinghai-Tibet Plateau was obtained from previous literature reports and local monitoring data, which provided the number and subtypes of isolates from all kinds of hosts, including people, rodents, fleas, etc. Because *M. himalayana* was the predominant host in the plague focus, we assumed that the subtypes of *Y. pestis* isolates infecting a marmot population were consistent with all subtypes in this area. We counted the total number of isolates and the number of different subtypes in different areas. The government conducted thorough same surveillance in the aforementioned regions, so we believe that the investigation accurately reflects the prevalence of plague in those areas. Furthermore, we compared the differences in *Y. pestis* subtypes between regions using the chi-square test for both biomarker clustering results.

### The relationship between genetic diversity and plague outbreaks

We included human plague outbreaks collected during 1954–2020 from the plague focus. Tang et al*.* [36] pointed out that the main infection pattern of human plague outbreaks during 1958—2021 was skinning and eating *M. himalayana* (130/198). Given that plague outbreaks in nature are affected by many other factors, such as environmental conditions [37], we selected the generalized linear mixed model (GLMM) to investigate the effect of genetic diversity (*Ho*) on plague outbreaks. The clustering results of microsatellites were set as random effects [plague outbreaks ~ *Ho* + (1|Cluster)]. A positive estimate would suggest a significant influence of marmot genetic diversity on plague outbreaks.

## Results

### Population genetics of microsatellites for *M. himalayana*

In this study, a total of 503 individual marmots collected from 12 populations were successfully genotyped at 13 pairs of fluorescent microsatellite loci []. All primers showed polymorphism (Table S3). A total of 162 alleles were detected, with a mean value of 12.462. The E locus had 16 alleles, with a large span and high genetic diversity. The R and S loci have the least number of alleles, with 9 alleles. The allele richness, representing the number of alleles standardized to the smallest sample size, was consistent with the number of alleles. The allelic richness of the E locus was the highest, reaching 15.996. The lowest richness was at the S locus, at 8.994. Polymorphic information content (PIC) was not consistent with the number and richness of alleles. In addition, the highest polymorphic information occurred at the M locus, up to 0.867. The R locus was relatively low at 0.681, showing moderate polymorphism. The genetic diversity of all populations was assessed using 13 pairs of microsatellite primers (Table 2). The number of alleles (*Na*) varied in different populations, ranging from 3.769 (QLC) to 8.769 (XHC). The number of effective alleles (*Ne*) in all populations was lower than the *Na*, with an average of 4.231, and most of them were between 3 and 4. The maximum mean *Ne* was recorded for the TRC population from Tongren County (3.083), and the minimum was recorded for QLC from Qilian County (3.093). The average Shannon’s information (*I*) was 1.561. The highest was still the TRC population (1.766), while the lowest QLC was only 1.167. The observed heterozygosity (*Ho*) was 0.499—0.808, with an average value of 0.723. The expected heterozygosity (*He*) was slightly higher than the *Ho*. Moreover, the highest was from the TRC population (0.784), followed by the XHC population (0.776). The lowest occurred in the QLC population (only 0.637). The fixation indices (*Fit*) of JZC, ZKC, and QLC were negative. The heterozygosity of these three populations was high, and the genetic differentiation was obvious. *Fit* values of the other 9 populations were positive, indicating high homozygosity and low genetic differentiation. No natural selection occurred because all populations were in Hardy–Weinberg equilibrium (HWE).

**Table 2:** Genetic diversity in 12 populations of *M. himalayana* in the Qinghai-Tibet Plateau

	N	*Na*	*Ne*	*I*	*Ho*	*He*	HWE	*Fit*	*Fis*
DQC	10	4.923	3.763	1.407	0.499	0.720	NS	0.308	0.059
AND	10	5.846	3.813	1.467	0.646	0.702	NS	0.062	0.454
GEM	23	7.077	4.016	1.535	0.699	0.707	NS	0.031	0.009
NQC	55	8.000	4.898	1.697	0.754	0.757	NS	0.011	0.539
XHC	60	8.769	4.865	1.750	0.773	0.776	NS	0.005	0.112
JZC	9	5.000	3.924	1.440	0.795	0.732	NS	-0.091	0.057
TRC	54	8.385	5.221	1.766	0.749	0.784	NS	0.052	0.454
ZKC	50	7.846	4.535	1.698	0.782	0.767	NS	-0.017	0.114
HZC	65	7.308	4.099	1.573	0.710	0.735	NS	0.035	0.107
WLC	113	8.231	4.598	1.675	0.745	0.763	NS	0.022	0.246
TJC	48	7.538	3.941	1.561	0.713	0.726	NS	0.018	0.106
QLC	6	3.769	3.093	1.167	0.808	0.637	NS	-0.247	0.040
Mean		6.891	4.231	1.561	0.723	0.734			

**Abbreviations**: *N* effective number of samples, *Na* number of alleles per population, *Ne* effective allele, *I* Shannonʼs information index, *Ho* observed heterozygosity, *He* expected heterozygosity, HWE Hardy–Weinberg equilibrium test, NS means populations were in HWE, *Fit* Fixation Index, *Fis* inbreeding coefficient

**Fig. 2:**
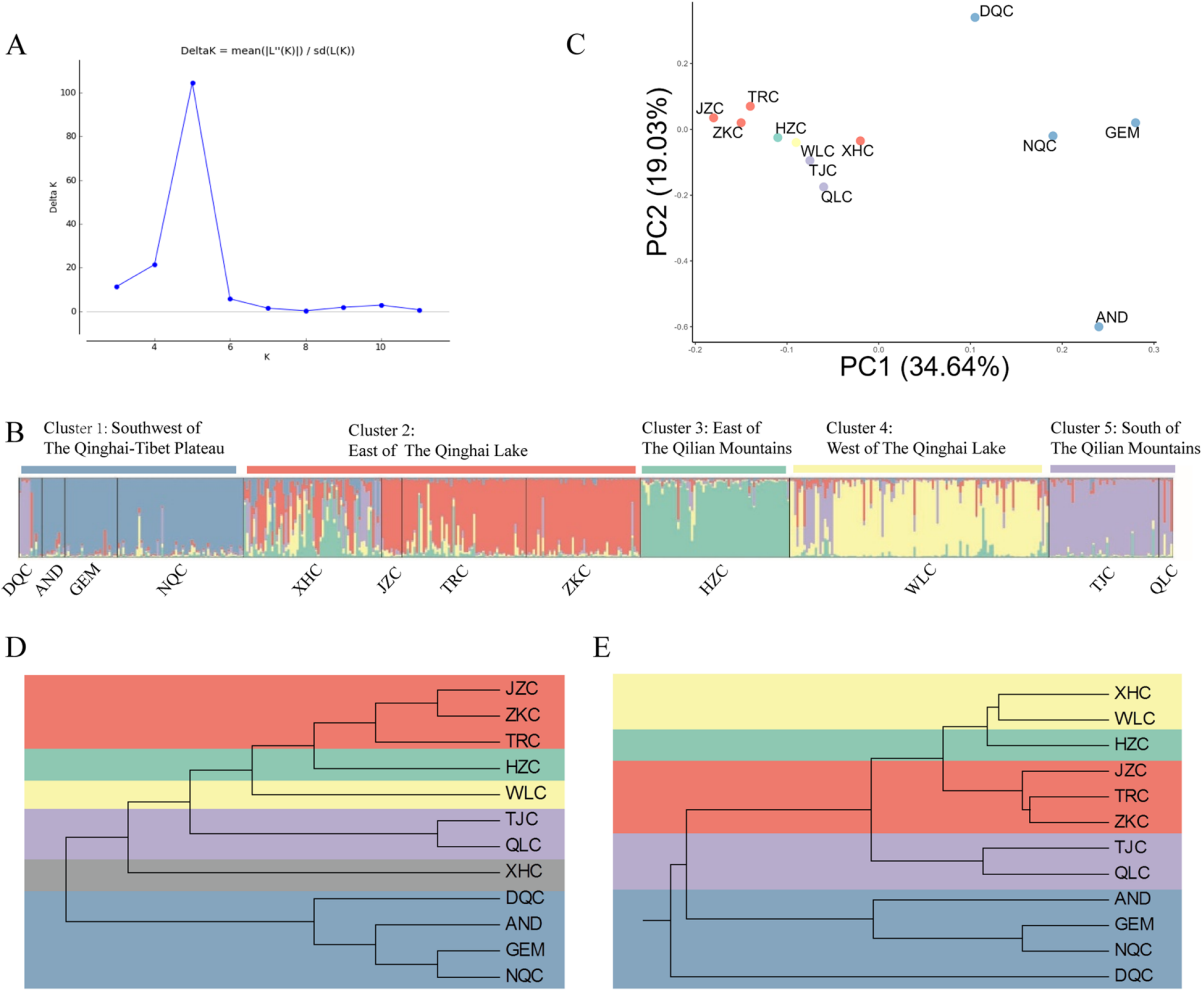
The clustering results based on microsatellites. **A** Plot of ΔK; the peak represents the most likely number of clusters (K = 5); **B** Bayesian clustering results inferred by STRUCTURE with the most likely model (K = 5). Each vertical bar represents an individual. The height of each bar indicates the probability of assignment to each of K optimal clusters; **C** PCoA plot showing genetic similarities among populations of *M. himalayana*; **D** NJ tree based on the genetic distance of all populations; **E** UPGMA dendrogram based on *Nei’s* standard genetic distance, showing the relationships between sampled cities. Blue, red, green, yellow and purple indicate Clusters 1–5, respectively

To reveal the genetic structure of marmot populations, we selected the Structure software that does not need to know its genetic background for the study. In addition, the ∆K curve was generated by Structure Harvester with the most likely number of clusters as 5 (Fig. 2A). The samples from DQC, AND, GEM, and NQC were in the first group (Cluster 1); XHC, JZC, TRC, and ZKC made up the second cluster (Cluster 2); and samples from HZC and WLC formed Clusters 3 and 4, respectively. The populations (TJC and QLC) belonged to Cluster 5 (Fig. 2B). The same result as structure clustering can be seen in the PCoA figure (Fig. 2C). We have two large clusters with Cluster 1 on the right and Cluster 2 on the left. The other three clusters are scattered around Cluster 2. A different cluster result was proposed by the NJ tree (Fig. 2D) and UPGMA tree (Fig. 2E). The result of UPGMA confirmed the result of Structure, while the NJ tree was closer to the distribution of the geographical region.

### Effects on the genetic divergence of *M. himalayana*

The *Fst* and *Nm* values are shown in Table 3. The paired *Fst* values in the *M. himalayana* distribution area were all less than 0.15, indicating that the *M. himalayana* populations in the Qinghai-Tibet Plateau are at the stage of low and moderate differentiation. Among them, the *Fst* value between DQC and QLC is the largest (0.121), which leads to the smallest *Nm* (1.815). The gene flow between TRC and ZKC was the highest (13.597), suggesting the most frequent genetic exchange between two populations of marmot in Tongren and Zeku counties of Huangnan Prefecture. The AMOVA results showed the source of variation (Table 4). The percentage of variation within individuals accounted for the largest, nearly 90%. The difference between different groups was less than 3.0%, but a significant *Fsc* value was detected, indicating a low degree of differentiation in *M. Himalaya.*

**Table 3:** *Fst* (lower left) and *Nm* (top right) matrix table of different marmot populations

	DQC	AND	GEM	NQC	XHC	JZC	TRC	ZKC	HZC	WLC	TJC	QLC
DQC		2.120	2.687	3.338	3.422	2.622	3.484	3.126	2.655	3.015	2.558	1.815
AND	0.105		3.607	4.933	3.590	2.094	2.679	2.466	2.548	3.031	2.440	2.042
GEM	0.085	0.065		9.631	3.335	2.216	2.763	2.630	2.350	2.870	2.750	1.932
NQC	0.070	0.048	0.025		5.854	2.804	4.450	3.754	3.715	5.026	3.838	2.882
XHC	0.068	0.065	0.070	0.041		4.720	10.444	8.689	9.041	11.187	6.305	4.177
JZC	0.087	0.107	0.101	0.082	0.010		7.884	7.608	4.172	5.817	4.023	2.672
TRC	0.067	0.085	0.083	0.053	0.023	0.031		13.597	9.465	11.584	5.067	3.031
ZKC	0.074	0.092	0.087	0.062	0.028	0.032	0.018		5.442	8.161	4.791	2.963
HZC	0.086	0.089	0.096	0.063	0.027	0.057	0.026	0.044		9.681	5.220	2.677
WLC	0.077	0.076	0.080	0.047	0.022	0.041	0.021	0.030	0.025		6.466	3.973
TJC	0.089	0.093	0.083	0.061	0.038	0.059	0.047	0.050	0.046	0.037		4.520
QLC	0.121	0.109	0.115	0.080	0.056	0.086	0.076	0.078	0.085	0.059	0.052

**Table 4:** Results of hierarchical analysis of molecular variance (AMOVA) for 13 microsatellite loci

Source of Variation	Sum of Squares	Variance Components	Percentage of Variation	Fixation indices
Among Groups	263.477	0.1511	2.818	Fct = 0.028
Among populations within groups	132.736	0.25754	4.804	Fsc = 0.048
Among individuals within the population	2503.455	0.15293	2.853	Fit = 0.104
Within individuals	2411	4.80122	89.559	Fis = 0.030
Total	5310.667	5.36094		

*Abbreviations*: Fct genetic differences among groups, Fsc genetic differences among population within groups, Fit genetic differences among populations

The Mantel test and MMRR showed consistent results (Table 5). The correlation between environmental variables and genetic distance was not significant (Mantel test: *P* = 0.1; MMRR: *P* = 0.279). In addition, the fit was better when the geographical variable was the Euclidean distance (Mantel test: *R*^*2*^ = 0.645, *P* < 0.001; MMRR: *R*^*2*^ = 0.645, *P* < 0.001) rather than LCP (Mantel test: *R*^*2*^ = 0.60, *P* < 0.01; MMRR: *R*^*2*^ = 0.60, *P* < 0.01), and the correlation between genetic variation and the geographical distance was always significant. Furthermore, a relatively high proportion of genetic variation could be explained when geographical variables and environmental data were combined (MMRR: *R*^*2*^ = 0.757, *P* < 0.001).

**Table 5:** Contribution of geographical distance and environmental variables to the genetic differentiation by Mantel test and MMRR

	Mantel test		MMRR	
	*R*^*2*^	*P*	*R*^*2*^	*P*
IBDe	0.645	0.01	0.645	0.001
IBDo	0.6	0.01	0.6	0.001
IBE	0.075	0.1	0.075	0.279
IBDe + IBE	/	/	0.757	0.001
IBDo + IBE	/	/	0.705	0.001

### Population genetics of mtDNA for *M. himalayana*

**Fig. 3:**
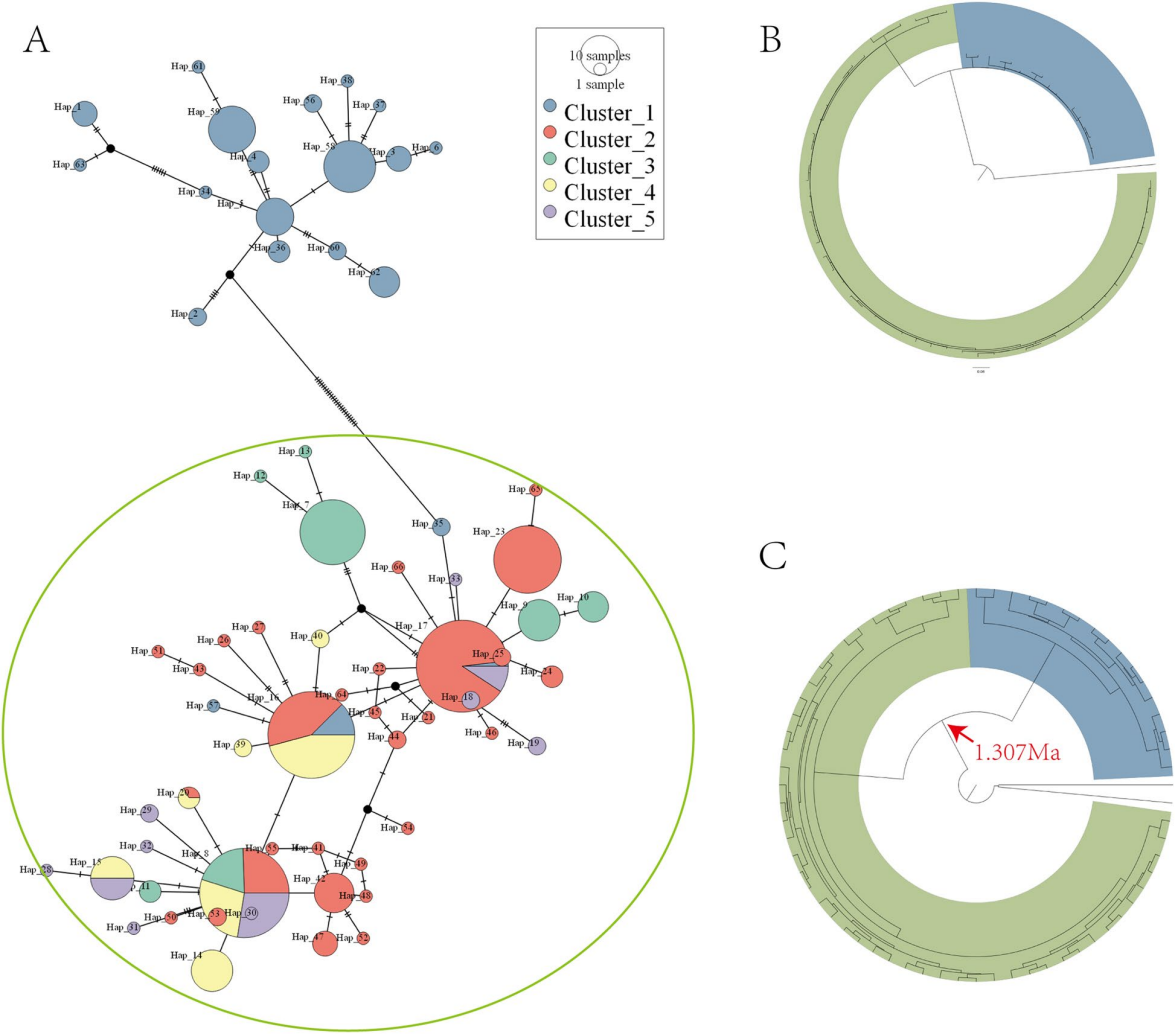
Phylogenetic results based on mtDNA. **A** The network plot based on 66 haplotypes of *M. himalayana* using the median-joining method. The size of each circle dictates the frequency of haplotypes, and the white dots represent missing haplotypes (not sampled or extinct). The pie charts are shared haplotypes. Mitochondrial phylogeny including Bayes analysis (**B**) and time tree (**C**) for *M. himalayana*. Two different background colors illustrate the two clusters (blue: south; green: north). Arrows indicate divergence time estimates at 1.307 Ma

We obtained 66 haplotypes from 389 concatenated mitochondrial DNA (mtDNA) sequences for *M. himalayana*. The haplotype diversity (Hd) was 0.932, and the nucleotide diversity index (π) was 0.007. The average number of nucleotide differences (k) was 11.39. Mitochondrial DNA showed high genetic diversity, which was consistent with microsatellite performance. The haplotype network diagram consisted of two parts, as shown in Fig. 3A. We used microsatellite-clustering results as the standard to conduct statistical analysis of haplotype frequencies. The top group of the network diagram corresponded to Cluster 1, which contained populations distributed in the south of the Qinghai-Tibet Plateau, while the bottom group was composed of other clusters from the northern Qinghai-Tibet Plateau. Phylogenetic inferences according to ML analyses (Fig. 3B) and molecular dating (Fig. 3C) showed similar classification results. Here, we visualized the branches with only two kinds of color highlights for display: blue for Group South and green for Group North. This phylogenetic result was consistent with the large-scale spatial distribution. In addition, the main diversification period between these two groups was approximately 1.307 Mya. The neutrality test unraveled the demographic history of mtDNA lineages of *M. himalayana* (Table S8). The significant statistics of both tests were only for TRC. This means that there was a history of population expansion, while no changes occurred in other populations.

### Molecular subtyping analysis of *Y. pestis* isolates

**Fig. 4:**
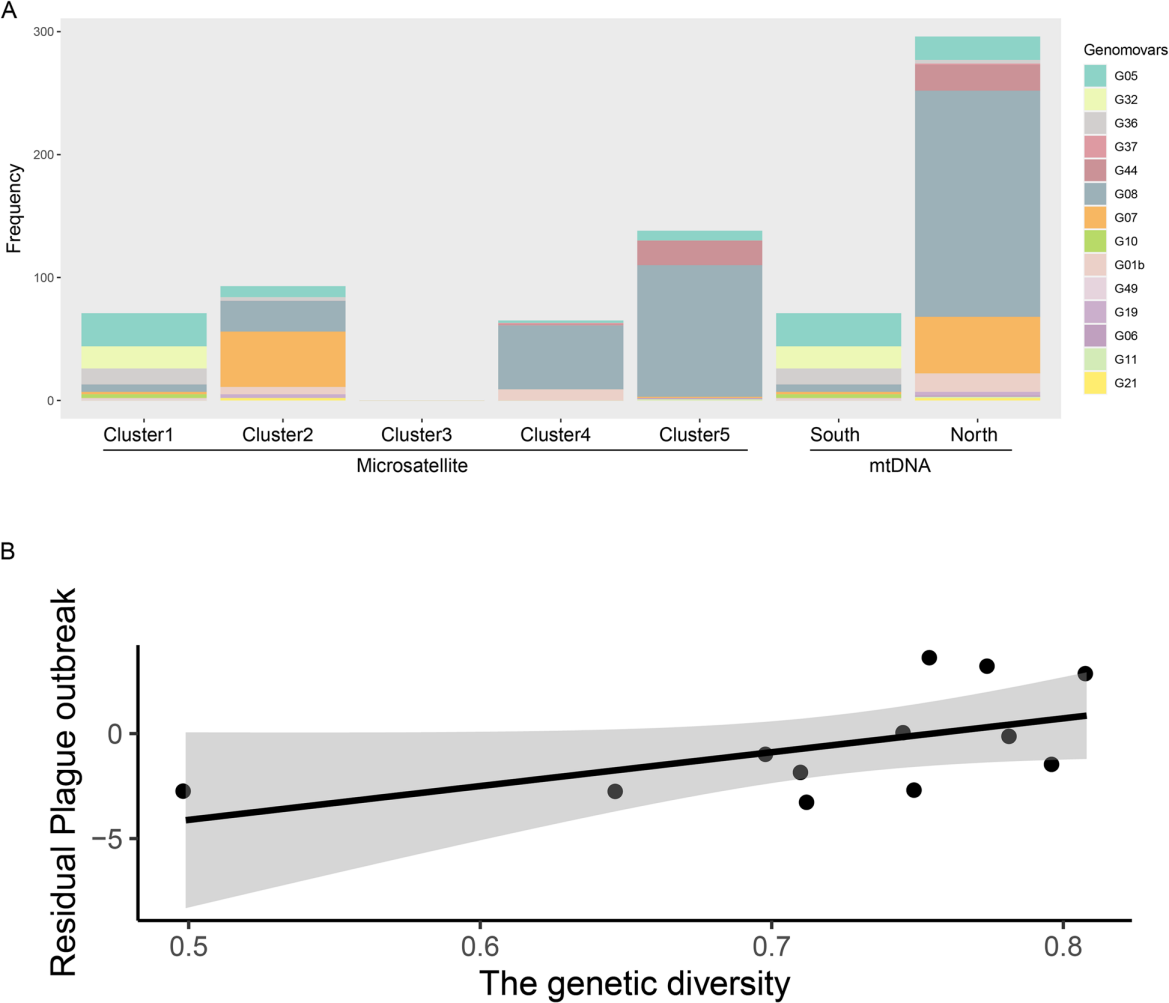
**A** Bar graphs indicating the frequency of *Y. pestis* DFR genomovars in the Qinghai-Tibet Plateau. Correlation plot between genetic diversity and human plague outbreaks/episodes. **B** Correlation scatter plot between the genetic diversity and residual plague outbreak for microsatellite clustering results. For the purpose of plotting, the residual plague outbreak for each site was calculated by using a mixed-effect generalized linear model to control for the random effect of clustering results (covariates in the statistical analysis are reported in the text). Each dot represents a population. The gray area represents the 95% confidence interval

A total of 367 isolates of *Y. pestis*, grouped into 14 genomovars, were included in this study [38–44] (Table S9). Seven genomovars comprised more than 10 strains, which covered 96.7% (355/367) of all strains. To our knowledge, there are no reports of human plague occurrence or isolation of DQC and HZC. The genomovars in different areas (Fig. 4A) exhibited a significant region-specific distribution. For instance, G32 was mostly found in GEM, G36 in NQC, and G44 mainly in QLC. Moreover, G05, G32, and G36 were endemic in the southern Qinghai-Tibet Plateau, while most trains from the north were identified as G08, G07, G44 and G01b. The chi-square test results for microsatellite and mtDNA clustering results were both significant (microsatellite: χ^2^ = 318.1, df = 39, *p* < 2.2e^−16^; mtDNA: χ^2^ = 204.52, df = 13, *p* < 2.2e^−16^; the significance level was set as 0.05), indicating significant differences in *Y. pestis* subtypes among regions.

### The relationship between genetic diversity and plague outbreaks

As expected, the population genetic diversity of *M. himalayan* was positively correlated with human plague outbreaks based on microsatellite clustering results (Fig. 4B: 23.72 ± 4.31, *p* < 0.001).

## Discussion

To our knowledge, this is the first study to investigate the population genetics of *M. himalayana* on such a large scale and correlate the clustering results of *M. himalayana* to the molecular subtype distribution of *Y. pestis*. The results revealed that *M. himalayana* populations could be divided into two main clusters located in the south and north of the Qinghai-Tibet Plateau. *Y. pestis* also showed a consistent geographical distribution, as indicated by the significant frequency differences of isolates in these two areas. This is also the first study to provide evidence that the increased genetic diversity of plague hosts is positively associated with plague outbreaks.

Genetic diversity is the foundational core of ecosystem and species diversity and is essential for species to sustain their evolutionary potential [45]. The number of alleles, ranging from 3.769 to 8.769, is significantly related to the sample size. It was advised that at least 30 individuals should be analyzed in most cases to detect all alleles [46]. In our study, there were few differences in the number of alleles among populations with large sample sizes (more than 30). Takezaki [47] proposed that the expected heterozygosity calculated by the microsatellite was between 0.3—0.8, indicating high population genetic polymorphism. Largely, *M. himalayana* populations had high genetic diversity, similar to other studies [48–50]. For mtDNA, we detected a total of 66 haplotypes, which had high haplotype diversity (0.932) but low nucleotide diversity. This phenomenon is common in rodents [51]. Nucleotide diversity caused by a single base mutation may take longer to accumulate than haplotype diversity.

According to recent syntheses of different studies, the Qinghai-Tibet Plateau Mountains reached their current elevations mainly between the late Miocene and late Pliocene. The uplift of the Qinghai-Tibet Plateau resulted in profound climatic and environmental changes in both the plateau region and Asia at large [52–54]. The genetic relationship of *M. himalayana* populations was closely related to its geographical distribution. The phylogenetic trees of *M. himalayana* were in line with the clustering results of other mammals [55–57]. *Phrynocephalus erythrurus* diverged into two major lineages/subspecies corresponding to the Northern and Southern Qiangtang Plateau composed of the Tanggula Mountains, Gangdisi Mountains, and Nyenqing Tanggula Mountains [58]. The population divergence of the Tibetan gazelle (*Procapra picticaudata*) was influenced by the uplift of the Qinghai-Tibet Plateau, whereas its dispersal capacity far exceeds that of muroid rodents [59]. The timing of the divergence (approximately 1.3 Ma) fell with the C stage of the Qinghai-Tibetan Movement, which changed the pattern of Hadley atmospheric circulation on the plateau. Many areas, especially the northern part, became rapidly arid, and some lakes began to disappear; for example, the Qaidam paleolake swiftly disappeared in the middle Pleistocene [54, 60].

The Qinghai-Tibet Plateau is dominated by mountain landforms and has a variety of landforms, such as mountain hills, hills, intermountain basins, alpine plains, rivers, riverbank terraces, lakes, valleys, wide valley beaches, and Gobi deserts [61]. Habitat selection of *M. himalayana* is mainly due to elevation, temperature, the presence of accumulative formations, and feeding conditions [62]. The geographical barrier, such as high elevation mountains and rivers, is the main reason that leads to the differentiation. All populations of Cluster 1 (Pop1-4: DQC, AND, GEM and NQC) were located in the south of Kunlun Mountain and Bayankla Mountain, which belongs to the southwest region of the Tibetan Plateau. HZC (Cluster 3, Pop9) lay to the east of the Qilian Mountains and was separated from the population of Huangnan prefecture (Pop6-8: JZC, TRC, and ZKC) by the Yellow River. Cluster 5 (Pop11-12: QLC, TJC) was located south of the Qilian Mountains and north of the Buha River. Mountains and rivers were formed by the Qing-Zang tectonic movements, which created geographic barriers that reduced the gene flow between isolated populations and promoted allopatric divergence, which often restricted the dispersal of animals, including frogs, lizards, birds, and particularly small mammals with weak diffusion capacity [63–68]. *Hanuman langurs* in the Nepal Himalaya region exhibit high genetic diversity, and their population genetic structure is strongly shaped by river barriers characterized by fast water flow and cold snow water [69]. Nevertheless, the fit of Euclidean distance is slightly stronger than that of LCP. A better fit of linear distance to genetic distance was found in Alpine marmots than along river valleys, indicating that *M. himalayana* may have the ability to climb mountains rather than just migrate along valleys and corridors. The mountains of the Qinghai-Tibet Plateau only increase migration distance but do not form a complete physical barrier.

According to the results of microsatellite data, XHC (Pop4) could be grouped in three different ways: 1) groups with the three populations in Huangnan Prefecture (JZC, TRC, and ZKC); 2) groups with WLC; and 3) independent groups. For mtDNA, XHC shared haplotypes with all four populations above. In this study, XHC and populations from Huangnan Prefecture tended to be grouped together. Although these two areas are separated by the Yellow River, the separation is not complete, and there are many roads and bridges connecting them. On the other hand, the Yellow River has often had dry seasons in its history. In addition, it has been reported that *M. himalayana* may have swimming ability. These factors increase the possibility of large-scale gene exchange and explain why the genetic structure of *M. himalayana* populations on the northern side of the Qiangtang Plateau is more pronounced and less differentiated.

The genetic divergence observed among populations in this study may be related to the geographical distribution of *M. himalayana*. Lower paired *Fst* values existed within the cluster, such as between GEM and NQC or between TRC and ZKC. *M. himalayana* in the Qinghai-Tibet Plateau is in the middle and low degree of differentiation stage (paired *Fst* < 0.25). The AMOVA results suggested that significant variances among clusters indicated genetic differences between different regions. We explored the main drivers of divergence in *M. himalayana*. The geographical distance was correlated with the genetic distance, which indicated that the geographical distance had a significant effect on the differentiation of *M. himalayana*. Meanwhile, we compared the contribution of these two kinds of geographical distances to divergence separately. The model fitted with the Euclidean distance is better than that fitted with LCP. This is mostly because physical barriers do not limit marmot migration. The influence of environmental factors on *M. himalayana* was far from significant, with *P* > 0.05, but increased the explanation of the degree of differentiation combined with geographical distance. Therefore, climate heterogeneity plays a role in the differentiation of *M. himalayana* but is not the main driving force.

Based on phylogenetic analysis results of mitochondrial genes, *M. himalayana* on the Qinghai-Tibet Plateau can only be divided into two lineages (Group South and Group Nouth), while the populations from Group North were further separated into four clusters. The clustering results showed obvious discordances between nuclear genetics and mtDNA, which could be interpreted by incomplete lineage sorting of ancestral polymorphisms (ILS) and different patterns of nuclear and mtDNA gene flow due to sex-biased dispersal [70, 71]. *M. himalayana* may exhibit a male-biased dispersal pattern similar to that of *Marmota monax* [72]. This was broadly supported by empirical evidence from mammals, in which males normally disperse further from their natal area. Extensive gene flow can also be reflected in the admixture analyses. Some individuals are observed to have genetic components from multiple clusters. Moreover, our study highlights the significance of incorporating both matrilineally and biparentally inherited markers in tracing the evolutionary history of a species, enhancing our understanding of its genetic dynamics.

Since *Y. pestis* was first isolated from *M. himalayana* in 1954, the plague focus of *M. himalayana* on the Qinghai-Tibet Plateau now covers > 443,290 km^2^. The *Y. pestis* genomic types in the focus were also numerous and complex. The phylogeny of Qinghai Plateau isolates of *Y. pestis* based on genetic markers showed that Group 1. IN2, the dominant population identified with SNPs, was divided into 3 subgroups (1. IN2A, 1. IN2B, 1. IN2C) using MLVA and CRISPR, revealing clear geographic clustering [73]. The majority of 1. IN2A strains were located in the southwest Qinghai Plateau. Three of the four 1. IN2A isolates near Qinghai Lake were collected from *M. himalayan* and are thought to be caused by the migration of this species. Subgroup 1. IN2C was mainly distributed encircling Qinghai Lake. Subgroup 1. IN2B was distributed in two separate regions, one for the southern foot of the Qilian Mountains and the other for the Huangnan region. This result can also prove that there may be some communication between marmots of Qilian County and the Huangnan region because the *Nm* value between them was larger. The same regional distribution result was obtained for *Y. pestis* by using only CRISPR in the Qinghai-Tibet Plateau [74]. In general, the results of different methods of *Y. pestis* genotype and geographical distribution indicate that there are at least three main types: 1) cluster in the southwest Tibetan Plateau located in the south of Bayankla Mountain; 2) cluster around Qinghai Lake; and 3) cluster in the Qilian Mountains. The result of clustering was similar to that of *M. himalayana*, and there were different classification results for different populations around Qinghai Lake, which may be due to more frequent gene exchanges in this area compared with other areas, as revealed by Table 3. Taken together, the consistency in the geographical distribution of *M. himalayana* and *Y. pestis* may be the result of local host‒pathogen coevolution. This phenomenon is widely observed in nature. Beth et al*.* [75] found that spatial clustering results of arctic foxes closely matched the distribution of rabies virus in Alaska, USA. In our study, it was supposed that the genetic variation during a long-term arms race between host defense and pathogen virulence was preserved due to the geographical separation caused by the uplift of the Qinghai-Tibet Plateau.

Pathogen prevalence can affect the spillover process [76]. An increase in animal plague outbreaks could expand the interface with pathogens, which in turn increases the risk of human plague outbreaks. The genetic diversity of *M. himalayana* had a significant positive association with human plague outbreaks, indicating that the risk of plague spillover increases as biodiversity increases. Our result was consistent with previous studies. Sun et al [77]. reported a positive relationship between rodent host species richness and human plague in China during the third pandemic. On the one hand, the high genetic diversity of the host population facilitated the maintenance of *Y. pestis* in natural foci, in which resistant individuals were able to provide refuge to *Y. pestis* and prevent the pathogen from local extinction [78]. In our study, we detected a strong relationship between the genetic diversity of *M. himalayana* and the number of *Y. pestis* subtypes. On the other hand, the high genetic diversity of *M. himalayana* may result from strong gene flow between different populations, which is beneficial to plague spread in *M. himalayana* populations. For instance, plague seroprevalence levels were significantly correlated with the genetic structure of rat populations in the Madagascan plague focus, suggesting that plague distribution is related to the effective dispersal of rats [79]. Furthermore, this process may accelerate the evolution of different variants of *Y. pestis*, which help the pathogen persist and increase human plague outbreaks [80, 81]. However, some studies concluded that increasing biodiversity is associated with a reduction in the risk of infectious diseases. Walsh et al [82]. found that species richness demonstrated strong positive associations with Kyasanur disease virus (KFDV) outbreaks, and this association could be substantially modified by forest loss. Civitello et al [83]. provided broad evidence that host diversity inhibits parasite abundance using a meta-analysis, indicating that biodiversity loss could increase human and wild diseases. Overall, the generality of the relationship between biodiversity and disease remains unresolved, but our results help to resolve one of the most contentious issues in infectious disease ecology. Specifically, we noted that there were no human plague case reports in Huzhu, while some were reported in the surrounding areas [84]. Population genetic analyses revealed that the *M. himalayana* population in Huzhu was clustered independently and showed relatively low genetic diversity compared to those from other regions where human plague occurred. Thus, it is essential to expand the scope of surveillance toward the composition of the *M. himalayana* population and the *Y. pestis* infection rate.

## Conclusions

This investigation combined the genetic structure of hosts and diseases, providing key insights into the effect of biodiversity on pathogen spillover. *M. himalayana* populations displayed obvious genetic structure resulting from the uplift of the Qinghai-Tibet Plateau. The phylogeographical distribution of *M. himalayana* is consistent with the distribution of *Y. pestis* subtypes. Human plague outbreaks can be positively related to the genetic diversity of the reservoir. This insight gained can improve our understanding of biodiversity for pathogen spillover and provide municipally directed targets for biodiversity-based One Health surveillance development, which will be an informative next step toward increased monitoring of *M. himalayana* dynamics and yielding the highest benefit from tailored intervention.

### Supplementary Information

**Supplementary Material 1. **

## Data Availability

The microsatellite data are deposited in Dryad: https://datadryad.org/stash/share/AHxjOO8qb2SaVA7r7RkR-9_KwFgA34VeLYq_LE_aMIE. DNA sequences were submitted to GenBank database under the accession numbers OR388130-OR388535 (cox1 gene) and OR389498-OR389973 (cytb gene).

## Abbreviations

AMOVA: Analysis of molecular variance
COI: Cytochrome c oxidase subunit 1 gene
Cytb: Cytochrome b
DFR: Different region
HWE: Hardy–Weinberg equilibrium
IBD: Isolation-by-distance
IBE: Isolation-by-environment
mtDNA: Mitochondrial DNA
LD: Linkage disequilibrium
NJ: Neighbor-joining
MMRR: Multiple matrix regression with randomization test
PCoA: Principal components analysis
PIC: Polymorphic information content
