# Supplementary material for: High genetic diversity of the himalayan marmot relative to plague outbreaks in the Qinghai-Tibet Plateau, China
Source: BMC Genomics. 2024 Mar 8;25:262. doi: 10.1186/s12864-024-10171-y (PMC10921737; doi:10.1186/s12864-024-10171-y)
Supplement: Supplementary file 1 — Supplementary Material 1. [file 12864_2024_10171_MOESM1_ESM.docx]

**High genetic diversity of the himalayan marmot relative to plague outbreak in the Qinghai-Tibet Plateau, China**

Ying Ma ^a,b,†^, Pengbo Liu ^b,†^, Ziyan Li^c^, Yujuan Yue ^b^, Yanmei Zhao ^a^, Jian He ^a^, Jiaxin Zhao ^b,d^, Xiuping Song^b^, Jun Wang ^b^, Qiyong Liu ^b^, Liang Lu ^b,*^

Table S.1 Sampling information of *M. himalayana* used in the present study

| No. | Pop | County | Region | Sample sizes | Longitude | Latitude | Elevation (m) |
| --- | --- | --- | --- | --- | --- | --- | --- |
| 1 | DQC | Deqin County, Diqing Prefecture, Yunnan | South | 10 | 99.26°E | 28.25°N | 4000-4200 |
| 2 | AND | Anduo County, Naqu City, Tibet | South | 10 | 91.68°E | 32.26°N | 4600-4800 |
| 3 | GEM | Ge’ermu County, Haixi Prefecture, Qinghai | South | 23 | 92.57°E | 34.21°N | 4400-4600 |
| 4 | NQC | Nangqian County, Yushu Prefecture, Qinghai | South | 55 | 96.15°E | 32.57°N | 3600-3800 |
| 5 | XHC | Xinghai Couty, Hainan Prefecture, Qinghai | North | 60 | 99.97°E | 35.27°N | 3800-4000 |
| 6 | JZC | Jianzha County, Huangnan Prefecture, Qinghai | North | 9 | 101.92°E | 35.94°N | 2200-2400 |
| 7 | TRC | Tongren County, Huangnan Prefecture, Qinghai | North | 54 | 102.00°E | 35.61°N | 3400-3600 |
| 8 | ZKC | Zeku County, Huangnan Prefecture, Qinghai | North | 50 | 101.52°E | 35.07°N | 4000-4200 |
| 9 | HZC | Huzhu County, Haidong Prefecture, Qinghai | North | 65 | 102.39°E | 36.92°N | 2400-2600 |
| 10 | WLC | Wulan County, Haixi Prefecture, Qinghai | North | 113 | 98.95°E | 36.98°N | 3200-3400 |
| 11 | TJC | Tianjun County, Haixi Prefecture, Qinghai | North | 48 | 98.80°E | 37.51°N | 3600-3800 |
| 12 | QLC | Qilian County, Haibei Prefecture, Qinghai | North | 6 | 100.24°E | 38.18°N | 2600-2800 |

Table S.2 The primer sequences for amplification of mitochondrial gene

| Primer | Sequences |
| --- | --- |
| COI | COI-B: 5'-CCTACTC(A/g)gCCATTTTACCTATg-3'  COI-R: 5'-ACTTCTgggTgTCCAAAgAATCA-3' |
| Cytb | Cytb-L1: 5'-ACCAATgACATgAAAAATCATCgTT-3'  Cytb-H1: 5'-TCTCCATTTCTggTTTACAAgAC-3' |

Table S. 3 19 biological environmental variables used in mantel test and MMRR for *M. himalayana*

| **Code** | **Description** | **Source** |
| --- | --- | --- |
| Bio1 | Annual Mean Temperature | http://www.worldclim.org, WorldClim 2004 |
| Bio2 | Mean monthly temperature range (Mean Diurnal Range) | http://www.worldclim.org, WorldClim 2004 |
| Bio3 | Isothermality (mean monthly temperature range /temperature annual range) (* 100) | http://www.worldclim.org, WorldClim 2004 |
| Bio4 | Temperature Seasonality (standard deviation * 100) | http://www.worldclim.org, WorldClim 2004 |
| Bio5 | Max Temperature of the Warmest Month | http://www.worldclim.org, WorldClim 2004 |
| Bio6 | Min Temperature of the Coldest Month | http://www.worldclim.org, WorldClim 2004 |
| Bio7 | Temperature Annual Range (BIO5 - BIO6) | http://www.worldclim.org, WorldClim 2004 |
| Bio8 | Mean Temperature of the Wettest Quarter | http://www.worldclim.org, WorldClim 2004 |
| Bio9 | Mean Temperature of the Driest Quarter | http://www.worldclim.org, WorldClim 2004 |
| Bio10 | Mean temperature of the warmest quarter | http://www.worldclim.org, WorldClim 2004 |
| Bio11 | Mean Temperature of the Coldest Quarter | http://www.worldclim.org, WorldClim 2004 |
| Bio12 | Annual precipitation | http://www.worldclim.org, WorldClim 2004 |
| Bio13 | Precipitation of the Wettest Month | http://www.worldclim.org, WorldClim 2004 |
| Bio14 | Precipitation of the driest month | http://www.worldclim.org, WorldClim 2004 |
| Bio15 | Precipitation Seasonality (Coefficient of Variation) | http://www.worldclim.org, WorldClim 2004 |
| Bio16 | Precipitation of the Wettest Quarter | http://www.worldclim.org, WorldClim 2004 |
| Bio17 | Precipitation of the Driest Quarter | http://www.worldclim.org, WorldClim 2004 |
| Bio18 | Precipitation of the Warmest Quarter | http://www.worldclim.org, WorldClim 2004 |
| Bio19 | Precipitation of the Coldest Quarter | http://www.worldclim.org, WorldClim 2004 |

Table S. 4 The environmental variables results of 12 sample sites

| Variables | DQC | AND | GEM | NQC | XHC | JZC | TRC | ZKC | HZC | WLC | TJC | QLC |
| --- | --- | --- | --- | --- | --- | --- | --- | --- | --- | --- | --- | --- |
| Bio1 | 0.29 | -2.97 | -0.26 | 1.05 | 5.43 | 4.41 | -0.38 | -0.45 | -3.53 | 2.45 | -2.73 | 10.15 |
| Bio2 | 14.7 | 14.0 | 13.9 | 10.5 | 13.6 | 14.3 | 14.3 | 15.2 | 15.1 | 14.2 | 13.2 | 11.1 |
| Bio3 | 35.5 | 36.8 | 36.1 | 30.4 | 35.4 | 37.0 | 37.9 | 39.5 | 39.0 | 40.7 | 37.7 | 41.9 |
| Bio4 | 973 | 880 | 912 | 855 | 878 | 854 | 843 | 800 | 895 | 759 | 840 | 577 |
| Bio5 | 19.2 | 14.1 | 17.2 | 17.2 | 22.5 | 21.5 | 16.2 | 15.9 | 14.3 | 18.1 | 13.1 | 22.1 |
| Bio6 | -22.3 | -23.9 | -21.2 | -17.4 | -15.8 | -17.0 | -21.4 | -22.6 | -24.6 | -16.7 | -22.0 | -4.4 |
| Bio7 | 41.4 | 38.1 | 38.4 | 34.6 | 38.3 | 38.5 | 37.6 | 38.5 | 38.8 | 34.8 | 35.1 | 26.4 |
| Bio8 | 11.5 | 7.5 | 10.4 | 11.1 | 15.6 | 14.3 | 9.4 | 8.8 | 7.1 | 11.3 | 7.2 | 16.8 |
| Bio9 | -11.2 | -13.0 | -11.0 | -10.0 | -5.0 | -6.8 | -10.4 | -9.8 | -13.8 | -6.5 | -11.4 | 3.8 |
| Bio10 | 11.5 | 7.5 | 10.4 | 11.1 | 15.6 | 14.3 | 9.4 | 8.8 | 7.1 | 11.3 | 7.2 | 16.8 |
| Bio11 | -12.4 | -14.1 | -11.9 | -10.0 | -6.1 | -6.8 | -11.3 | -10.9 | -14.7 | -7.3 | -13.3 | 2.8 |
| Bio12 | 391 | 319 | 255 | 442 | 446 | 489 | 404 | 566 | 277 | 514 | 428 | 765 |
| Bio13 | 96 | 77 | 60 | 96 | 97 | 104 | 94 | 120 | 78 | 114 | 113 | 150 |
| Bio14 | 1 | 1 | 0 | 1 | 0 | 1 | 1 | 2 | 1 | 2 | 3 | 7 |
| Bio15 | 109.1 | 104.7 | 105.0 | 96.6 | 96.0 | 94.8 | 100.7 | 94.0 | 116.3 | 101.3 | 114.5 | 73.3 |
| Bio16 | 256 | 210 | 168 | 259 | 255 | 277 | 248 | 321 | 193 | 314 | 291 | 381 |
| Bio17 | 4 | 5 | 2 | 5 | 4 | 5 | 6 | 9 | 5 | 10 | 9 | 30 |
| Bio18 | 256 | 210 | 168 | 259 | 255 | 277 | 248 | 321 | 193 | 314 | 291 | 381 |
| Bio19 | 4 | 6 | 3 | 5 | 4 | 5 | 6 | 9 | 6 | 11 | 9 | 42 |
| elev | 2793 | 4000 | 3462 | 2963 | 2452 | 2768 | 3665 | 3697 | 4519 | 3900 | 4728 | 2679 |
| PET | 3620 | 3818 | 2579 | 4425 | 3791 | 4377 | 3590 | 5886 | 2587 | 4571 | 3935 | 5726 |

Table S. 5 The correlation matrix table of 21 environment variables

|  | elev | Bio9 | Bio8 | Bio7 | Bio6 | Bio5 | Bio4 | Bio3 | Bio2 | Bio19 | Bio18 | Bio17 | Bio16 | Bio15 | Bio14 | Bio13 | Bio12 | Bio11 | Bio10 | Bio1 | PET |
| --- | --- | --- | --- | --- | --- | --- | --- | --- | --- | --- | --- | --- | --- | --- | --- | --- | --- | --- | --- | --- | --- |
| elev | 1.00 |  |  |  |  |  |  |  |  |  |  |  |  |  |  |  |  |  |  |  |  |
| Bio9 | -0.60 | 1.00 |  |  |  |  |  |  |  |  |  |  |  |  |  |  |  |  |  |  |  |
| Bio8 | -0.88 | 0.87 | 1.00 |  |  |  |  |  |  |  |  |  |  |  |  |  |  |  |  |  |  |
| Bio7 | 0.12 | -0.77 | -0.43 | 1.00 |  |  |  |  |  |  |  |  |  |  |  |  |  |  |  |  |  |
| Bio6 | -0.60 | 0.97 | 0.86 | -0.83 | 1.00 |  |  |  |  |  |  |  |  |  |  |  |  |  |  |  |  |
| Bio5 | -0.91 | 0.78 | 0.98 | -0.25 | 0.75 | 1.00 |  |  |  |  |  |  |  |  |  |  |  |  |  |  |  |
| Bio4 | 0.12 | -0.82 | -0.45 | 0.92 | -0.81 | -0.31 | 1.00 |  |  |  |  |  |  |  |  |  |  |  |  |  |  |
| Bio3 | 0.30 | 0.40 | 0.04 | -0.37 | 0.27 | 0.02 | -0.62 | 1.00 |  |  |  |  |  |  |  |  |  |  |  |  |  |
| Bio2 | 0.37 | -0.53 | -0.44 | 0.76 | -0.68 | -0.28 | 0.50 | 0.32 | 1.00 |  |  |  |  |  |  |  |  |  |  |  |  |
| Bio19 | -0.20 | 0.82 | 0.48 | -0.90 | 0.82 | 0.35 | -0.92 | 0.60 | -0.51 | 1.00 |  |  |  |  |  |  |  |  |  |  |  |
| Bio18 | -0.25 | 0.75 | 0.48 | -0.68 | 0.69 | 0.39 | -0.81 | 0.48 | -0.36 | 0.73 | 1.00 |  |  |  |  |  |  |  |  |  |  |
| Bio17 | -0.16 | 0.82 | 0.46 | -0.90 | 0.80 | 0.32 | -0.94 | 0.62 | -0.50 | 0.99 | 0.80 | 1.00 |  |  |  |  |  |  |  |  |  |
| Bio16 | -0.25 | 0.75 | 0.48 | -0.68 | 0.69 | 0.39 | -0.81 | 0.48 | -0.36 | 0.73 | 1.00 | 0.80 | 1.00 |  |  |  |  |  |  |  |  |
| Bio15 | 0.66 | -0.88 | -0.77 | 0.68 | -0.86 | -0.69 | 0.78 | -0.25 | 0.54 | -0.73 | -0.68 | -0.72 | -0.68 | 1.00 |  |  |  |  |  |  |  |
| Bio14 | -0.03 | 0.71 | 0.33 | -0.88 | 0.71 | 0.18 | -0.89 | 0.60 | -0.48 | 0.96 | 0.80 | 0.98 | 0.80 | -0.60 | 1.00 |  |  |  |  |  |  |
| Bio13 | -0.23 | 0.76 | 0.48 | -0.71 | 0.70 | 0.38 | -0.82 | 0.51 | -0.37 | 0.77 | 0.99 | 0.83 | 0.99 | -0.67 | 0.84 | 1.00 |  |  |  |  |  |
| Bio12 | -0.41 | 0.86 | 0.62 | -0.74 | 0.81 | 0.53 | -0.87 | 0.46 | -0.44 | 0.81 | 0.97 | 0.85 | 0.97 | -0.84 | 0.81 | 0.96 | 1.00 |  |  |  |  |
| Bio11 | -0.64 | 0.99 | 0.89 | -0.75 | 0.98 | 0.80 | -0.81 | 0.35 | -0.54 | 0.79 | 0.73 | 0.79 | 0.73 | -0.90 | 0.68 | 0.74 | 0.85 | 1.00 |  |  |  |
| Bio10 | -0.88 | 0.87 | 1.00 | -0.43 | 0.86 | 0.98 | -0.45 | 0.04 | -0.44 | 0.48 | 0.48 | 0.46 | 0.48 | -0.77 | 0.33 | 0.48 | 0.62 | 0.89 | 1.00 |  |  |
| Bio1 | -0.77 | 0.96 | 0.97 | -0.62 | 0.95 | 0.91 | -0.66 | 0.22 | -0.50 | 0.67 | 0.64 | 0.65 | 0.64 | -0.87 | 0.53 | 0.64 | 0.78 | 0.98 | 0.97 | 1.00 |  |
| PET | -0.29 | 0.62 | 0.39 | -0.56 | 0.56 | 0.31 | -0.73 | 0.34 | -0.33 | 0.60 | 0.90 | 0.66 | 0.90 | -0.75 | 0.65 | 0.87 | 0.91 | 0.62 | 0.39 | 0.54 | 1.00 |

Table S. 6 The environment distance matrix table of 12 sampling sites

|  | DQC | AND | GEM | NQC | XHC | JZC | TRC | ZKC | HZC | WLC | TJC | QLC |
| --- | --- | --- | --- | --- | --- | --- | --- | --- | --- | --- | --- | --- |
| DQC | 0.000 |  |  |  |  |  |  |  |  |  |  |  |
| AND | 2.037 | 0.000 |  |  |  |  |  |  |  |  |  |  |
| GEM | 0.854 | 1.282 | 0.000 |  |  |  |  |  |  |  |  |  |
| NQC | 1.267 | 2.742 | 1.943 | 0.000 |  |  |  |  |  |  |  |  |
| XHC | 1.784 | 3.305 | 2.508 | 0.576 | 0.000 |  |  |  |  |  |  |  |
| JZC | 1.781 | 2.773 | 2.273 | 0.679 | 0.822 | 0.000 |  |  |  |  |  |  |
| TRC | 1.781 | 0.717 | 1.279 | 2.180 | 2.718 | 2.110 | 0.000 |  |  |  |  |  |
| ZKC | 2.977 | 2.000 | 2.709 | 2.763 | 3.125 | 2.313 | 1.479 | 0.000 |  |  |  |  |
| HZC | 3.188 | 1.461 | 2.334 | 4.123 | 4.697 | 4.222 | 2.171 | 3.197 | 0.000 |  |  |  |
| WLC | 3.194 | 2.012 | 2.854 | 3.056 | 3.433 | 2.625 | 1.587 | 0.319 | 3.094 | 0.000 |  |  |
| TJC | 3.363 | 1.326 | 2.593 | 3.965 | 4.504 | 3.865 | 1.787 | 2.264 | 1.210 | 2.083 | 0.000 |  |
| QLC | 7.250 | 7.024 | 7.405 | 6.245 | 6.094 | 5.575 | 6.406 | 5.044 | 8.215 | 5.120 | 7.149 | 0.000 |

Table S.7 Polymorphic information at 13 microsatellite loci used

| locus | No. | *N* | *r* | PIC |
| --- | --- | --- | --- | --- |
| B | 503 | 12 | 12.000 | 0.842 |
| C | 503 | 13 | 12.994 | 0.805 |
| W | 502 | 11 | 10.996 | 0.801 |
| D | 500 | 10 | 10.000 | 0.792 |
| H | 501 | 14 | 13.994 | 0.797 |
| T | 500 | 13 | 13.000 | 0.701 |
| E | 502 | 16 | 15.996 | 0.840 |
| G | 503 | 15 | 14.994 | 0.864 |
| I | 503 | 12 | 11.994 | 0.814 |
| J | 503 | 14 | 13.994 | 0.818 |
| R | 503 | 9 | 9.000 | 0.681 |
| S | 503 | 9 | 8.994 | 0.766 |
| M | 502 | 14 | 13.996 | 0.867 |
| Mean |  | 12.462 | 12.458 | 0.799 |

Abbreviations: *N*, the number of alleles; *r*, allele richness; PIC, polymorphic information content;

Table S.8 The neutrality test results of *M. himalayana* based on mtDNA

| Pop | Tajima's D | *P* value | Fu's Fs | *P* value |
| --- | --- | --- | --- | --- |
| DQC | 1.33727 | 0.947 | 6.90612 | 0.995 |
| AND | 1.13663 | 0.874 | 0.72366 | 0.647 |
| GEM | 1.35612 | 0.918 | 3.23171 | 0.913 |
| NQC | -0.18239 | 0.46 | -1.16705 | 0.262 |
| XHC | 0 | 1 | 0 | N.A. |
| JZC | 2.01187 | 0.975 | 2.19722 | 0.823 |
| TRC | -1.64624 | 0.041* | -5.70029 | **0.002** |
| ZKC | -1.48747 | 0.053 | -2.4713 | 0.075 |
| HZC | -0.28843 | 0.425 | 2.28908 | 0.875 |
| WLC | -1.48195 | 0.057 | -8.91246 | **0.001** |
| TJC | -0.04077 | 0.575 | 5.64593 | 0.959 |
| QLC | -0.33203 | 0.408 | 0.14342 | 0.584 |

Table S.9 Information from 367 *Y. pestis* isolates in the Qinghai-Tibet Plateau from 1954 - 2021

| Pop | Period | Human plague ourbreak | Human plague cases | Total Isolates number | Isolates of  *M. himlayana* | G05 | G32 | G36 | G37 | G44 | G8 | G07 | G10 | G01b | G49 | G19 | G06 | G11 | G21 |
| --- | --- | --- | --- | --- | --- | --- | --- | --- | --- | --- | --- | --- | --- | --- | --- | --- | --- | --- | --- |
| DQC | 1954 - 2021 | 0 | 0 | 0 | 0 | 0 | 0 | 0 | 0 | 0 | 0 | 0 | 0 | 0 | 0 | 0 | 0 | 0 | 0 |
| AND | 1954 - 2021 | 0 | 0 | 7 | 7 | 3 | 2 | 0 | 0 | 0 | 0 | 2 | 0 | 0 | 0 | 0 | 0 | 0 | 0 |
| GEM | 1967 - 2006 | 2 | 5 | 28 | 16 | 9 | 16 | 0 | 0 | 0 | 2 | 0 | 0 | 0 | 1 | 0 | 0 | 0 | 0 |
| NQC | 1973 - 2004 | 13 | 40 | 36 | 24 | 15 | 0 | 13 | 0 | 0 | 4 | 0 | 3 | 1 | 0 | 0 | 0 | 0 | 0 |
| XHC | 1956 - 2009 | 16 | 91 | 15 | 5 | 0 | 0 | 0 | 0 | 0 | 13 | 0 | 0 | 0 | 0 | 0 | 0 | 0 | 2 |
| JZC | 1954 - 1991 | 3 | 9 | 26 | 19 | 3 | 0 | 1 | 0 | 0 | 4 | 15 | 0 | 2 | 0 | 1 | 0 | 0 | 0 |
| TRC | 1954 - 1991 | 1 | 1 |  |  |  |  |  |  |  |  |  |  |  |  |  |  |  |  |
| ZKC | 1954 - 1991 | 6 | 22 |  |  |  |  |  |  |  |  |  |  |  |  |  |  |  |  |
| HZC | 1954 - 2021 | 0 | 0 | 0 | 0 | 0 | 0 | 0 | 0 | 0 | 0 | 0 | 0 | 0 | 0 | 0 | 0 | 0 | 0 |
| WLC | 1966 - 2011 | 5 | 5 | 65 | 44 | 2 | 0 | 0 | 1 | 1 | 52 | 0 | 0 | 9 | 0 | 0 | 0 | 0 | 0 |
| TJC | 1957 - 2009 | 3 | 17 | 34 | 25 | 2 | 0 | 0 | 0 | 0 | 32 | 0 | 0 | 0 | 0 | 0 | 0 | 0 | 0 |
| QLC | 1956 - 2011 | 24 | 35 | 104 | 77 | 6 | 0 | 0 | 0 | 20 | 75 | 1 | 0 | 0 | 0 | 0 | 1 | 1 | 0 |
